# Imposed visual feedback delay of an action changes mass perception based on the sensory prediction error

**DOI:** 10.3389/fpsyg.2013.00760

**Published:** 2013-10-23

**Authors:** Takuya Honda, Nobuhiro Hagura, Toshinori Yoshioka, Hiroshi Imamizu

**Affiliations:** ^1^Cognitive Mechanisms Laboratories and Brain Information Communication Research Laboratory Group, Advanced Telecommunications Research Institute InternationalKyoto, Japan; ^2^Research Fellow of the Japan Society for the Promotion of ScienceTokyo, Japan; ^3^Institute of Cognitive Neuroscience, University College LondonLondon, UK; ^4^Center for Information and Neural Networks, National Institute of Information and Communications Technology and Osaka UniversityOsaka, Japan

**Keywords:** sensorimotor prediction, feedback delay, mass perception, delay adaptation, prediction error

## Abstract

While performing an action, the timing of when the sensory feedback is given can be used to establish the causal link between the action and its consequence. It has been shown that delaying the visual feedback while carrying an object makes people feel the mass of the object to be greater, suggesting that the feedback timing can also impact the perceived quality of an external object. In this study, we investigated the origin of the feedback timing information that influences the mass perception of the external object. Participants made a straight reaching movement while holding a manipulandum. The movement of the manipulandum was presented as a cursor movement on a monitor. In Experiment 1, various delays were imposed between the actual trajectory and the cursor movement. The participants' perceived mass of the manipulandum significantly increased as the delay increased to 400 ms, but this gain did not reach significance when the delay was 800 ms. This suggests the existence of a temporal tuning mechanism for incorporating the visual feedback into the perception of mass. In Experiment 2, we examined whether the increased mass perception during the visual delay was due to the prediction error of the visual consequence of an action or to the actual delay of the feedback itself. After the participants adapted to the feedback delay, the perceived mass of the object became lighter than before, indicating that updating the temporal prediction model for the visual consequence diminishes the overestimation of the object's mass. We propose that the misattribution of the visual delay into mass perception is induced by the sensorimotor prediction error, possibly when the amount of delay (error) is within the range that can reasonably include the consequence of an action.

## Introduction

While performing an action, information on the timing of the sensory feedback has a crucial role in detecting the causal link between the action and its consequence (Kitazawa et al., 1995; Blakemore et al., 1999; Farrer et al., 2008; Tanaka et al., 2011; Honda et al., 2012a,b). For example, when the visual feedback is delayed, a self-generated visual motion is perceived as generated by someone else (Blakemore et al., 1999; Farrer et al., 2008). Furthermore, the motor learning process is also disrupted in such situations, possibly due to the failure of accurately linking the feedback information with one's own action (Kitazawa et al., 1995; Tanaka et al., 2011; Honda et al., 2012a,b). It has been suggested that the central nervous system (CNS) uses a forward model to predict the sensory consequence of an action (e.g., the position of the hand at a certain time point) by using a copy of the motor command (Miall et al., 1993; Wolpert et al., 1995; Miall et al., 2007). In such a scenario, the amount of prediction error, which is the difference between the predicted and the actual sensory feedback, contributes to detecting whether the sensory input is actually generated by the person.

The feedback timing information is not only used for linking the action and the consequence but can also contribute to the perception of the external environment. For example, the perception of a somatosensory event induced by self-touch is modified when a delay is imposed between the action and the touch (Blakemore et al., 1999). Likewise, it has been shown that delaying the visual feedback of an action while carrying an object makes people feel that the mass of that object is greater (Di Luca et al., 2011). Such evidence suggests that delay in the sensory feedback of an action may violate the authorship of the sensory consequence and, at the same time, change the quality of perception of that sensory event. In this study, we focus on the influence of feedback timing on the perceptual quality of the object's mass. Similar to the violation of authorship induced by the prediction error, in this case, the difference in the visually predicted hand position and the actual visual feedback (prediction error) may also contribute to such an overestimation of the object's mass. However, it is not yet clear whether the prediction error or the actual delay itself plays the major role in causing this phenomenon.

To test these two possibilities, we set up a reaching experiment where participants made a straight reaching movement while holding a manipulandum. The movement of the manipulandum was presented as a cursor movement on a monitor, which allowed us to impose various delays between the actual hand movement and the visual cursor movement. In Experiment 1, we examined the relationship between the amount of delay and the illusory increase of mass. Since the authorship of the sensory consequence is violated with a longer imposed delay between the action and the consequence, (Farrer et al., 2008) we predict that the mass of the manipulandum will be perceived as heavier for a shorter delay but not for a longer delay.

In Experiment 2, we investigated the effect of delay adaptation on the perceived mass. If the prediction error were the cause of the increase in perceived mass, reducing the prediction error by adapting to the delay would alleviate the overestimation of the mass. On the other hand, if the actual delay were the cause, the mass would be overestimated irrespective of the adaptation.

## Materials and methods

### Participants

A total of 24 neurologically normal right-handed (Oldfield, 1971) volunteers (22 males and 2 females; age range, 20–44 years) participated. The study was approved by the Ethics Committee of the Advanced Telecommunications Research Institute. Written informed consent was obtained from all participants prior to performing the experiments.

### Apparatus

**Figure 1:**
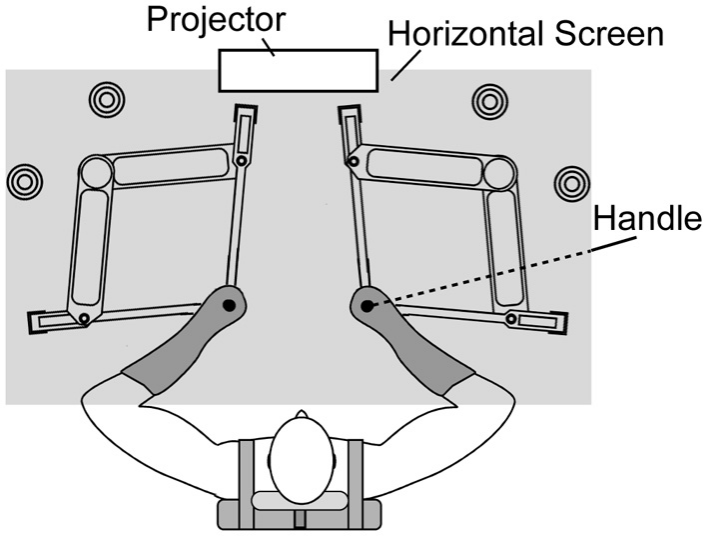
**Top-down view of twin visuomotor and haptic interface system (TVINS).** In Experiment 1, only the right-hand manipulandum was used. In Experiment 2, both manipulanda were used. The horizontal screen is illustrated as if it were transparent in order to show the manipulandum. In reality, it was opaque and reflected images generated by the projector installed above.

Participants sat on an adjustable chair while grasping the handle of a twin visuomotor and haptic interface system (TVINS) (Figure 1). The participant's forearm was secured to a support beam on the horizontal plane. TVINS consists of two parallel-linked, direct-drive floating manipulanda using air magnets. Thus, the experiment can be conducted either by using only one manipulandum or by using both at the same time. Each manipulandum was powered by two DC direct-drive motors controlled at 2000 Hz. TVINS yielded a virtual mass (*M*) according to the equation of motion: *M* = F/a. Here *F* is a resistance force generated by TVINS in proportion to the handle acceleration (*a*). We confirmed that the accuracy in online measurement of the acceleration was ± 0.04 m/s^2^ even at the peak acceleration. We also confirmed by measuring the resistance force with a spring scale that TVINS could generate a target force with the precision of 0.1 Kgf. The position of the manipulandum was measured using optical joint position sensors (4,800,000 pulse/rev). The position of the hand (handle of the manipulandum) was projected on a horizontal screen placed above the mechanical plane and below shoulder level. The projector refresh rate was 75 Hz. The screen prevented the participants from directly seeing their arm.

It should be noted that there was a slight time delay for the actual handle movement to be reflected as the cursor movement on the screen, due to the limitations of the computer's data processing speed. When the delay between the handle and the cursor movements was measured 10 times by a high-speed camera (600 Hz), it was found to be 42.5 ms (SD 2.4 ms) when around the handle position near the body (around the “starting position” in the experiments) and 41.8 ms (SD 2.4 ms) when at a distance from the body (around the “target position”). No significant difference between the positions [*t*_(18)_ = 2.11, *p* = 0.543] was observed. Since this delay is comparable across positions, and our interest was in the difference between the conditions, we believe that this delay itself will not affect our results. In the following, we describe the delay from this “baseline delay” but note that an additional 42-ms delay always existed in all of the conditions.

### Experiment 1

We tested how the difference in imposed visual delay between the actual movement and the cursor feedback information influences the mass perception.

#### Task procedure

Fourteen volunteers participated. By making a reaching movement while grasping the manipulandum with their right hand, participants moved the cursor toward the target presented 10 cm from the starting point on the screen. After the reaching movement, the handle automatically moved back to the starting position. Participants judged the perceived mass of the manipulandum after each trial.

Three target locations were prepared. The middle target was straight ahead from the starting point, and the other two were 20° rotated clockwise or counterclockwise around the starting point from the middle target's path. The peak velocity of the reaching movement was required to be within the range of 300–450 mm/s. A warning message appeared on the screen if the movement velocity of the handle was faster (“Fast”) or slower (“Slow”) than the set velocity range. The mass of the manipulandum was varied from trial to trial by adding a resistive force against the movement of the hand (see above). Nine mass values were prepared: 1, 2, 3, 4, 5, 6, 7, 8, and 9 kg. Furthermore, a variable delay was imposed between the cursor movement and the actual movement of the hand in each trial, where this delay was chosen from five values: 0, 100, 200, 400, or 800 ms. The experiment investigated every combination between mass and delay (9 masses × 5 delays: 45 combinations). Each combination was repeated 15 times in random order. Consequently, each participant carried out a total of 675 trials. The experiment was divided into five 135-trial blocks, and the participants were allowed to take a break between the blocks. After each reaching movement, participants judged whether the mass of the manipulandum was greater or smaller than the average of all of the mass values presented in the previous trials. This is a version of the “method of single stimuli,” (Morgan et al., 2000) which requires participants to use their internal criterion for the judgment. The accuracy of this method is comparable to, and even more accurate than, (Nachmias, 2006) the method that always presents a standard stimulus as a comparison stimulus (Wearden and Ferrara, 1995; Hagura et al., 2012). Moreover, this method enables us to increase the number of trials for a given period of time, which is crucial for reconstructing the psychometric function. The judgment (“lighter” or “heavier”) was made by pressing one of two buttons with the left hand. Before the experiment, participants practiced and experienced each mass 30 times in order to familiarize themselves with the distribution of the input mass.

#### Data analysis

Participants' judgments of the masses were analyzed separately for five imposed delays. Logistic regression was used to relate the percentage of “heavier” judgment to overall stimulus mass value for each participant. The form of the function was

$$y=\frac{1}{1+e^{\left(\frac{x-\alpha }{\theta }\right)}},$$

where α is the mass value corresponding to the point of subjective equality (PSE, the 50% response level on the psychometric function) and θ provides an estimate of the mass discrimination sensitivity. To estimate the parameters, the logistic function was fitted to the judgment data of individual subjects by using a generalized linear mode as implemented in a MATLAB *glmfit* function (MathWorks, Natick, MA, USA). One-Way analysis of variance (ANOVA) with repeated measures was used to test the effect of the delay value on both the PSE and the discrimination sensitivity. Ryan's multiple comparison tests were used to compare the 0-ms delay condition with the other delay conditions. The threshold for statistical significance was set at *p* < 0.05 throughout this study.

### Experiment 2

In Experiment 1, we found that the perceived mass of the manipulandum significantly increases with the amount of imposed visual feedback delay when the delay is in the short range, but not when it is in the longer range (see Results). Next, we examined whether decreasing the prediction error for the feedback delay would change this delay-induced overestimation of the object's mass.

#### Task procedure

Ten volunteers participated. There were two conditions: *delay condition* and *no-delay condition*. In *delay condition*, participants were continuously exposed to the visual feedback delay when reaching to a target with the manipulandum, whereas delay was not imposed in *no-delay* condition (*simple reach* trials). Between these reaching trials, the participants' ability to perceptually recognize the delay (*delay awareness* trials) and their perception of the manipulandum mass (*mass comparison* trials) were measured.

The two conditions were performed by each participant on two separate days. The order of the conditions was randomly assigned to each participant: five of them performed *no-delay condition* first, while the others performed *delay condition* first. Each condition consisted of 404 trials, which were divided into four 101-trial blocks. Each block consisted of 87 *simple reach* trials, 5 *delay awareness* trials, and 9 *mass comparison* trials. Participants took short breaks between blocks. Note that the *delay awareness* and *mass comparison* trials were identical between conditions. Therefore, any conditional difference observed in these trials would be due to the pre-exposure to the feedback delay occurring in the *simple reach* trials. The details of these different trial types are explained below.

In *simple reach* trials, participants made a right-hand reaching movement by moving the manipulandum toward the target that appeared 15 cm from the starting position. The visual feedback was delayed for 200 ms in *delay condition*, whereas no delay was imposed in *no-delay condition*. The aim of *simple reach* trials was to allow participants to adapt to the 200-ms delay in *delay condition* (and the lack of delay in *no-delay condition*). To maintain participants' concentration, in one of 10 to 11 trials, the target jumped to the 20° clockwise-rotated position immediately after the onset of the reaching. The *simple reach* trials were distributed pseudo-randomly in a block, where more than one *simple reach* was conducted before *delay awareness* or *mass comparison* trials.

**Figure 2:**
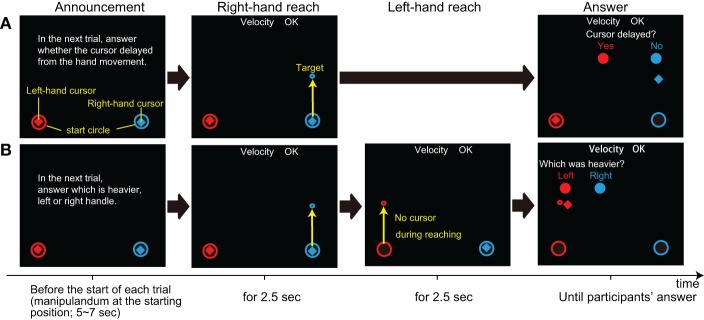
**Two types of trials in Experiment 2.** The horizontal flow is the sequence of each trial; **(A)** *Delay awareness* trial in which subjects were asked if they felt any delay in cursor movements, and **(B)** *mass comparison* trials in which subjects were asked to judge whether the right- or left-hand movement was heavier. The instruction was on the screen from the end of the last trial until the onset of the next trial (target appearance). The yellow letters and arrows are used to explain each display, but they are not shown on the screen during the actual experiment.

In *delay awareness* trials (sequence **A** in Figure 2), after making the right-hand reaching movement, participants were required to answer whether they felt any delay between their hand and the cursor movement. This trial was used to assess the change in perceptual sensitivity to the delay. Since we found in our pilot study that the delay of 200 ms was easily detectable, the cursor delay in *delay awareness* trials was set to 150 ms to avoid any ceiling effect.

Finally, in *mass comparison* trials (see sequence **B** in Figure 2), after making a right-hand reaching movement, participants were asked to make the same straight reaching movement with their left hand. Then, they were asked to judge whether the right hand was heavier or lighter than the left hand. The cursor delay was set to 200 ms for the right-hand movement, and there was no cursor presented for the left-hand movement. The mass value of all of the right-hand reaches was set to 3 kg (this was also the case for *simple reach* and *delay awareness*), while the mass was set to 1, 3, or 5 kg for left-hand reaches. This trial was used to evaluate the perception of mass under the delay of visual feedback, in the same manner used in Experiment 1. Since our aim was to extend our findings in Experiment 1, which was performed with the right hand, the left hand was used only to present the reference mass for the right hand.

Before *delay awareness* and *mass comparison* trials, participants were instructed about which type of trial they were going to perform (see “announcement” in Figure 2).

#### Data analysis

For the *delay awareness* and the *mass comparison* trials, the probability of judging the trial as “delayed” and that of judging the mass of the right hand “heavier” were calculated. These values were compared between the *delay* and *no-delay conditions*.

## Results

### Experiment 1

**Figure 3:**
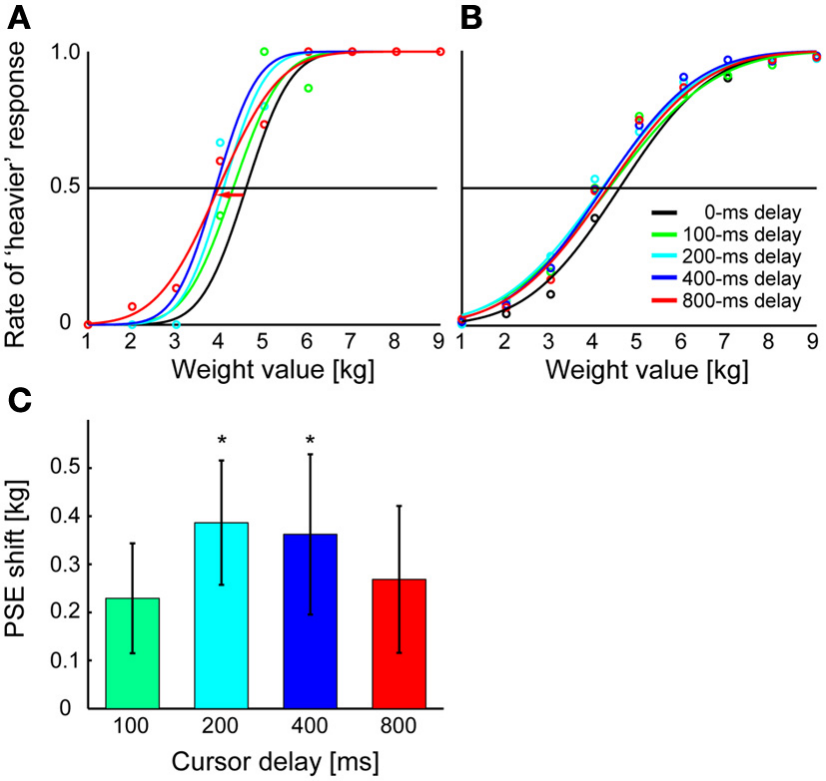
**Results of Experiment 1. (A)** Results are shown for a typical participant. The mass value at which each curve crosses the 0.5 line is PSE for each delay value. The red arrow indicates the shift of PSE for a 800-ms delay from that for a 0-ms delay (see panel** C**). **(B)** Psychometric functions are fitted to data averaged across participants. Average judgment rate across participants was calculated for each mass value, and sigmoid functions were fitted to the averaged rates. **(C)** For each delay, the shift of PSE from that for a 0-ms delay is shown. Shifts were calculated for each cursor delay and averaged across participants. Error bars indicate standard error of measures across participants. *p < 0.05 according to Ryan's multiple (four) comparison tests for difference in PSE between 0-ms delay and the other delay conditions.

The psychometric function in Figures 3A,B describes the participants' judgment of mass as a function of actually delivered mass. Figure 3A shows the psychometric function constructed for different imposed delays (0, 100, 200, 400, or 800 ms) of a representative participant, while Figure 3B shows that of the data averaged across all participants. One-Way ANOVA with repeated measures performed on the PSEs of different delay values showed a significant effect of delay on mass perception [*p* = 0.024, *F*_(4, 52)_ = 3.082]. Figure 3C shows a shift in PSE for each delay from the case of 0-ms delay. The *post hoc* comparison performed from the 0-ms delay condition showed that the PSE significantly shifted toward the heavier side when the 200-ms delay (*p* = 0.030 after correction with Ryan's nominal significant level) or the 400-ms delay (*p* = 0.038 after the correction) was imposed, but not when the delay was 100 ms (*p* = 0.175 after the correction) or 800 ms (*p* = 0.175 after the correction). Moreover, One-Way ANOVA with repeated measures performed on the discrimination sensitivity of different delay values showed no significant effect of delay [*p* = 0.130, *F*_(4, 52)_ = 1.866; mean sensitivity (±SD) was 0.97 ± 0.09 for a 0-ms delay, 1.11 ± 0.10 for a 100-ms delay, 1.02 ± 0.10 for a 200-ms delay, 0.94 ± 0.08 for a 400-ms delay, and 1.01 ± 0.08 for a 800-ms delay]. This indicates that sensitivity to the mass did not differ according to the delays. The results show that the visual feedback delay significantly modifies the mass perception of the manipulandum but failed to reach significance for a longer delay.

### Experiment 2

One participant was excluded from the analysis based on his extremely slow reaction times, that is, initiation of the movement onset from the target appearance was more than 1000 ms on average, possibly due to a lack of concentration on the task.

**Figure 4:**
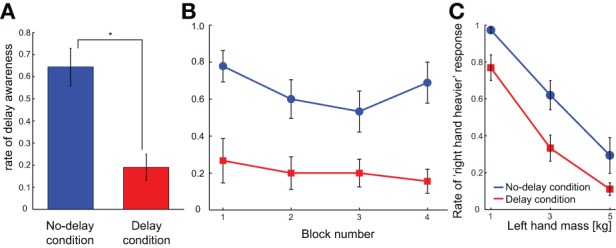
**Results of Experiment 2. (A)** Average delay awareness across participants is shown in each condition. *p < 0.05 according to the paired *t*-test. **(B)** Rate of the delay awareness is shown as a function of block number. **(C)** Average judgment of “right hand heavier” across participants is shown for each right-hand mass value in each condition. Error bars indicate standard error of measures across participants.

For the *delay awareness* trials, the rate of delay awareness was significantly higher in the no-delay condition than in the delay condition according to the paired *t*-test [*p* < 0.001; *t*_(8)_ = 6.468; Figure 4A]. Namely, participants tended to more accurately perceive the imposed 150-ms delay in the *no-delay condition* compared to the *delay condition*. This indicates that repeated exposure to the delay in the simple *reach trials* made the participants less sensitive to the delay. The lower sensitivity to the delay after being exposed to the delay was already observed in the first block of trials, and it continued throughout the experiment (Figure 4B). When we analyzed the data with a Two-Way ANOVA, using the effect of block number along with the effect of condition (*delay* or *no-delay*), only a main effect of the condition [*p* = 0.0001, *F*_(1, 8)_ = 47.059] was observed, without any main effect of the block [*p* = 0.102, *F*_(3, 24)_ = 2.313] nor of the interaction between the two factors [*p* = 0.592, *F*_(3, 24)_ = 0.649]. These results show that exposure to the delay seems to have an immediate impact on the delay sensitivity, and the effect was consistent throughout Experiment 2.

Following this tendency, participants perceived the mass of the manipulandum as lighter in the mass comparison trials of the *delay condition* compared to that of the *no-delay condition* (Figure 4C). Two-Way ANOVA with repeated measures showed significant main effects of the condition [*p* = 0.002, *F*_(1, 8)_ = 19.139] and the mass value [*p* < 0.001, *F*_(2, 16)_ = 71.627], without any significant effect of interaction [*p* = 0.558, *F*_(2, 16)_ = 0.605]. This indicates that the adaptation to the delay induced the insensitivity to the delay, and this was accompanied by the perception of smaller mass compared to when there was no adaptation. In other words, the perceived delay may play a critical role in judging the mass of an object while making a movement.

## Discussion

We examined how imposing a delay between an action and its visual feedback influences mass perception. In Experiment 1, participants felt that the manipulandum was heavier as the feedback delay increased to 400 ms, but this effect was less clear when the delay was 800 ms (Figure 3C). This indicates that mass perception modified by feedback delay is not solely related to the amount of delay. The results of Experiment 2 show that the mass overestimation was alleviated when the participants adapted to the delay, compared to when there was no adaptation (Figure 4C). This suggests the sensory feedback prediction error may play an important role in inducing the overestimation of mass.

Delaying the visual feedback during manual actions causes a discrepancy between visual and proprioceptive positional estimates of the hand, or between expected and actual hand positions. This kind of discrepancy tends to be attributed to the mass perception, making the participants feel that the hand-held object is heavier than expected (Di Luca et al., 2011). Within the range of the delay investigated in the previous study (0–200 ms), the perceived mass linearly increased as the delay increased. However, this was not the case for much longer delays, which was specifically tested in our experiment (Figure 3C); when the delay was 800 ms, the effect of overestimating the mass became variable. This shows that longer feedback delay is processed differently from shorter delays. A previous study showed that delaying the timing of a sensory consequence of an action makes people feel that the time between an action and its sensory consequence is shorter than it actually is (Haggard et al., 2002). This binding effect was regarded as an implicit measure of whether the sensory input is actually processed as one's own action (authorship of the sensory event) (Haggard et al., 2002; Haggard and Clark, 2003). Several studies have shown that the binding effect is modulated by temporal contiguity: When the feedback delay is large, the binding effect becomes weak (Haggard et al., 2002; Heron et al., 2009). Many studies have demonstrated that such binding does not occur if the delay is more than 200–300 ms (Blakemore et al., 1999; Haggard et al., 2002; Heron et al., 2009). In considering this evidence, the reason why the longer feedback delay (800 ms) was not reflected as an increase in mass may be due to the disruption of the association between an action and its sensory consequence: The longer delay may have violated the authorship of the sensory feedback information rather than being processed as the consequence of the action. Violation of action-authorship modifying the quality of the sensory perception may reflect findings in the literature showing that the participant's perceived intensity (amount of force) (Shergill et al., 2003) or the quality (ticklishness) of a tactile input depends on the applied timing of the tactile stimulus in relation to the participant's own action (Blakemore et al., 1999).

It should be noted that the violation of the authorship of the feedback information in the present study can occur without depending on the amount of delay; since the average movement time of reaching movement was 882 ± 88 ms, participants may not have related the 800-ms-delay feedback to their own movement simply because the movement had nearly terminated. Our current experimental design cannot separate the effects of these two factors, and so further study is needed to separate these possibilities.

In Experiment 2, when the participants were repeatedly exposed to the delay (*delay condition*), they became less sensitive to the delay compared to when not exposed to the delay (*no-delay condition*) (Figures 4A,B). Reduced sensitivity to the temporal delay shows that the participants were perceptually adapted to the feedback delay in the *delay condition*, as has been shown both in the perceptual domain (Haggard et al., 2002; Haggard and Clark, 2003) and in the motor control domain (Honda et al., 2012a,b). Accompanying this adaptation effect, the illusory increase in mass was significantly alleviated in the *delay condition* in comparison to the *no-delay condition* (Figure 4C; red plots are significantly below blue ones). This result clearly shows that the mass overestimation accompanying feedback delay is not caused by the actual delay itself, since the actual delay is constant in the two conditions (Figure 4C). Furthermore, this suggests that the factors changing in accordance with the perceptual temporal adaptation might be tightly linked to the alleviation of mass overestimation.

Two different types of adaptation may underlie the temporal adaptation observed between the action and the sensory input in this study. One is the adaptation between different sensory inputs, such as between vision and proprioception (Kambara et al., 2013). Feedback delay will lead to a discrepancy between the two, which may require calibration. The other possibility is the involvement of a motor command, providing prediction about the timing of the sensory reafference. In this case, adaptation may have occurred between the predicted and the actual timing of the incoming sensory input (prediction error). Either mechanism could have worked in our experiment. However, Stetson et al. (2006) showed that the strength of calibration of perceived timing between pressing a button and a visual flash is much weaker when the button press was replaced by a passive button touch. Other studies on delay perception have also suggested that prediction in the sensorimotor system is critical for a change in temporal perception (Haggard and Clark, 2003; Stetson et al., 2006). Therefore, we believe that the increase in mass perception dominantly involves motor-based prediction error. In any case, further study is necessary to clarify this point.

In conclusion, we propose that the misattribution of a visual delay to the increased mass perception is induced by the sensorimotor prediction error, and it seems to preferentially occur when the delay is within the range that can be attributed to the consequence of the action.

### Conflict of interest statement

The authors declare that the research was conducted in the absence of any commercial or financial relationships that could be construed as a potential conflict of interest.
